# Knowledge mapping of surgical smoke from 2003 to 2022: a bibliometric analysis

**DOI:** 10.1007/s00464-023-10641-6

**Published:** 2024-01-16

**Authors:** Chuang Li, Meng Geng, Shujun Li, Xianglan Li, Huiqin Li, Hufang Yuan, Fengxia Liu

**Affiliations:** 1 The Fourth Hospital of Hebei Medical University, Shijiazhuang, China; 2 Hebei Medical University Third Hospital, Shijiazhuang, China

**Keywords:** Surgical smoke, Bibliometric analysis, Knowledge map, VOSviewer, CiteSpace

## Abstract

**Purpose:**

The purpose of this study is to identify and characterize the literature on surgical smoke, visualize the data and sketch a certain trending outline.

**Methods:**

In the Web of Science Core Collection (WoSCC), all the data were acquired from January 1st 2003 to December 31st 2022. VOSviewer and CiteSpace were employed to visualize data, based on publications, bibliographic coupling, co-citation, or co-authorship relations. Microsoft Excel 2019 was used to comb and categorize all the statistics.

**Result:**

A total 363 of journal papers were retrieved. The publication number was in a slow but steady growth between 2003 and 2019, followed by a sharp surge in 2020, and then the publication kept in a productive way. Surgical endoscopy and other interventional techniques was the most active journal on surgical smoke. USA played an important role among all the countries/regions. There were 1847 authors for these 363 papers, among whom 44 authors published more than three articles on surgical smoke. “Surgical smoke”, “covid-19” and “surgery” were the top 3 appeared keywords, while the latest hot-spot keywords were “COVID-19”, “virus”, “transmission”, “exposure” and “risk”. There were 1105 co-cited references and 3786 links appeared in all 363 articles. Among them, 38 references are cited more than 10 times. The most co-cited article was “Detecting hepatitis B virus in surgical smoke emitted during laparoscopic surgery.” Based on the titles of references and calculated by CiteSpace, the top 3 cluster trend network are “laparoscopic surgery”, “COVID-19 pandemic” and “surgical smoke”.

**Conclusion:**

According to bibliometric analysis, the research on surgical smoke has been drawing attention of more scholars in the world. Increasing number of countries or regions added in this field, and among them, USA, Italy, and China has been playing important roles, however, more wide and intense cooperation is still in expectation.

**Supplementary Information:**

The online version contains supplementary material available at 10.1007/s00464-023-10641-6.

Surgical smoke is gaseous by-product generated by electrosurgical devices in cutting or coagulating tissues when operation is proceeding. The electrosurgical devices usually refer to electric cautery, bipolar forceps, ultrasonic scalpel, laser or plasma knife etc. They are so convenient and effective that surgical smoke is ubiquitous during almost every surgery in operating room, many unpleasant odors troubling and haunting around surgical staff. Many studies have shown that surgical smoke is hazard to health. It contains particulate matters 2.5 and mutagenetic or poisonous volatile organic compound [1–4], but rather, active virus and tumor [5–8]. Furthermore, studies have reported that surgical smoke from different tissue varies, and so it does with different electrosurgical devices [9–11].

As the extensively usage of electrosurgical devices, the adverse effect of surgical smoke become more obvious. Many methods have been introduced to decrease the ambient volume of smoke, like direct suction near the surgical site with suction apparatus, instant clearance of eschar adhered to the knives, opening 1 channel for dispersion in laparoscopic surgeries, smoke evacuator with filter in the neighborhood, electrostatic precipitation, and many improvement based on upper mentioned methods [12–15].

Bibliometric analysis is a statistical and mathematical measurement method about the quality and quantity of articles and publications. It analyzes certain topic or domain in journals, countries, funding agencies, keywords, contribution or cooperation of authors and ultimately gives a comprehensive overview as well as study trending for readers [16–18]. VOSviewer and CiteSpace are both bibliometric tools, which visualize the selected papers related to certain field based on co-author, co-occurrence, countries/regions cooperation, and so on. The former can be quick to create a bibliometric network graph, where Network Visualization, Overlay Visualization, and Density Visualization presenting the relationship and centrality of different items [19]. CiteSpace is a functionally designed bibliometric tool. It focuses on cluster-labeling, node-adjusting, paper information reviewing, and topic-extracting in LLR, LSI, MI algorithm [20]. CiteSpace is helpful to reveal new technologies, hot spots and trends, as well as explore the key paths and frontier developments in scientific research fields [21].

There are literature reviews [1, 22–25] or systematic reviews [26, 27] about surgical smoke, however, no bibliometric analysis had been done to give a qualitatively and quantitatively analyzing panorama related to surgical smoke. Therefore, this paper uses two kinds of bibliometric software—VOSviewer and CiteSpace, in order to identify and characterize the English literature on surgical smoke, visualize the data in the latest 20 years and sketch a certain trending outline in the field, helping the following researchers with potential topic and hot spots.

## Materials and methods

### Data collection

In the Web of Science Core Collection (WoSCC), all the data were searched and acquired from January 1st 2003 to December 31st 2022. Retrieved method and strategies were used as [TS = (“surgical smoke” OR “surgical plume” OR “surgical aerosol” OR “surgical particle” OR “surgical gas” OR “surgical smog”)]. The inclusion criteria were as follows: (1) peer-reviewed published original articles; (2) reviews; (3) language in English, meanwhile we excluded meeting abstracts, news items, letters, editorial materials, corrections, and other languages articles.

**Fig. 1:**
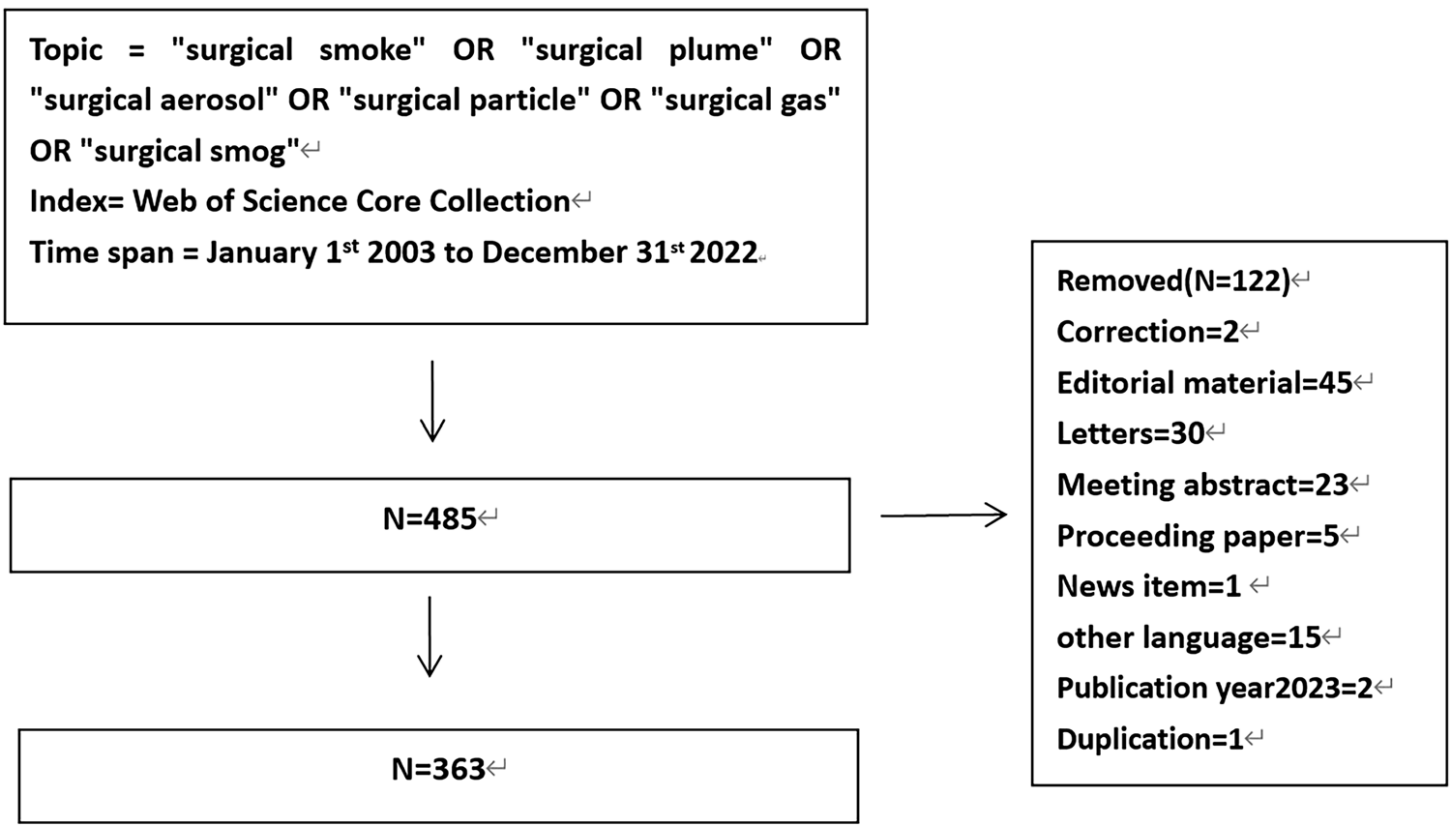
Flow chart of data collection

Totally 485 articles were obtained before 122 ones excluded. At last, we get 363 eligible publications, including 299 articles and 64 reviews (Fig. 1). All “Full Record and Cited References” of the data were exported in format “.xls” and “.txt” for further analysis.

### Data analysis

Bibliometric software CiteSpace 6.2.4 Advanced [28] and VOSviewer version 1.6.19 [29] were used to construct and visualize data, based on citation, bibliographic coupling, co-citation, or co-authorship relations. We used Microsoft Excel 2019 to comb, count, and categorize all the statistics, then displayed in chart.

## Results

### Annual publications and citations

**Fig. 2:**
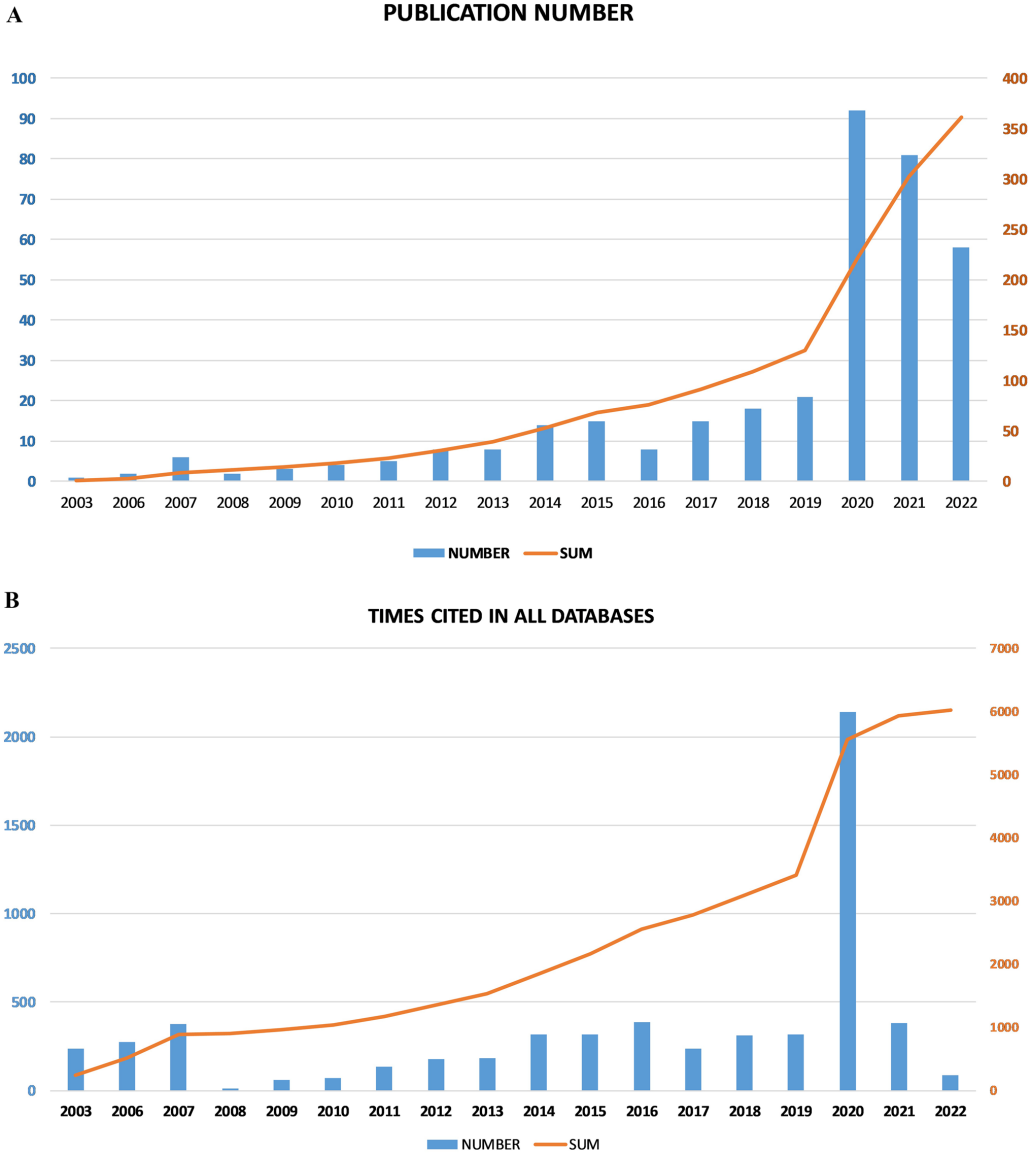
**A** Publications in the past 20 years. **B** Time cited in the past 20 years

From Fig. 2A, we could see the publication number of each year was in a slow but steady growth between 2003 and 2019 without data collected in 2004 and 2005. However, 2020 witnessed the sharp surge with the top publication number up to 92, still more than the following 2 years’ counts as were 80 and 58. This was due to the pandemic of Corona Virus Disease 2019 (COVID-19), which had been testified to spread via aerosol. Consequently, surgical smoke drew the experts’ attention to protecting medical staff from infecting in each surgery of COVID-19 patients. All of this was also reflected in Fig. 2B about citation—2020 was still at the peak with 2143 citations while the number in other years fluctuated around 100–300 except 2008, 2009, 2010, 2022 (below 100). The number of 2020 occupied more than 1/3 of the total citation number (*n* = 6024) between 2003 and 2022.

### Journal analysis

**Fig. 3:**
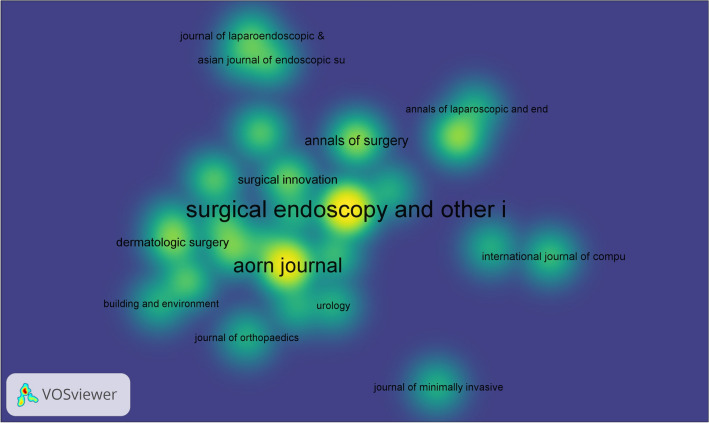
Density visualization of journals. Colors range from blue to green to yellow. The closer the color of the point to yellow, the higher the item weighs (Color figure online)

During 2003 to 2022, 205 journals have published articles on surgical smoke. We pick out the top ten journals among them as is shown in Table 1 and Fig. 3. Surgical endoscopy and other interventional techniques (SURG ENDOSC, IF:3.5), with 22 papers on its list, is the most active journal in this field. SURG ENDOSC is a publication of Springer which helps meet different patients’ needs. The third one Annals of Surgery (8 publications, IF:10.3) has the highest IF out of the top ten, which provides the international medical community with information on significant contributions to the advancement of surgical science and practice. These three journals published 89 articles, covering nearly a quarter of all the eligible data (*n* = 363).

**Table 1:** Publication number and impact factor of the top ten most-published journals

Rank	Journal	Number	Average impact factor (in past 5 years)
1	Surgical Endoscopy and Other Interventional Techniques	22	3.5
2	AORN Journal	18	1.1
3	Annals of Surgery	8	10.3
4	Surgical Laparoscopy Endoscopy & Percutaneous Techniques	8	1.2
5	Journal of Occupational and Environmental Hygiene	6	2.5
6	Surgical Innovation	6	1.7
7	ANZ Journal of Surgery	6	1.8
8	British Journal of Surgery	5	8.2
9	Dermatologic Surgery	5	2.7
10	International Journal of Environmental Research and Public Health	5	4.799

### Contributions of countries/regions to global publication

**Fig. 4:**
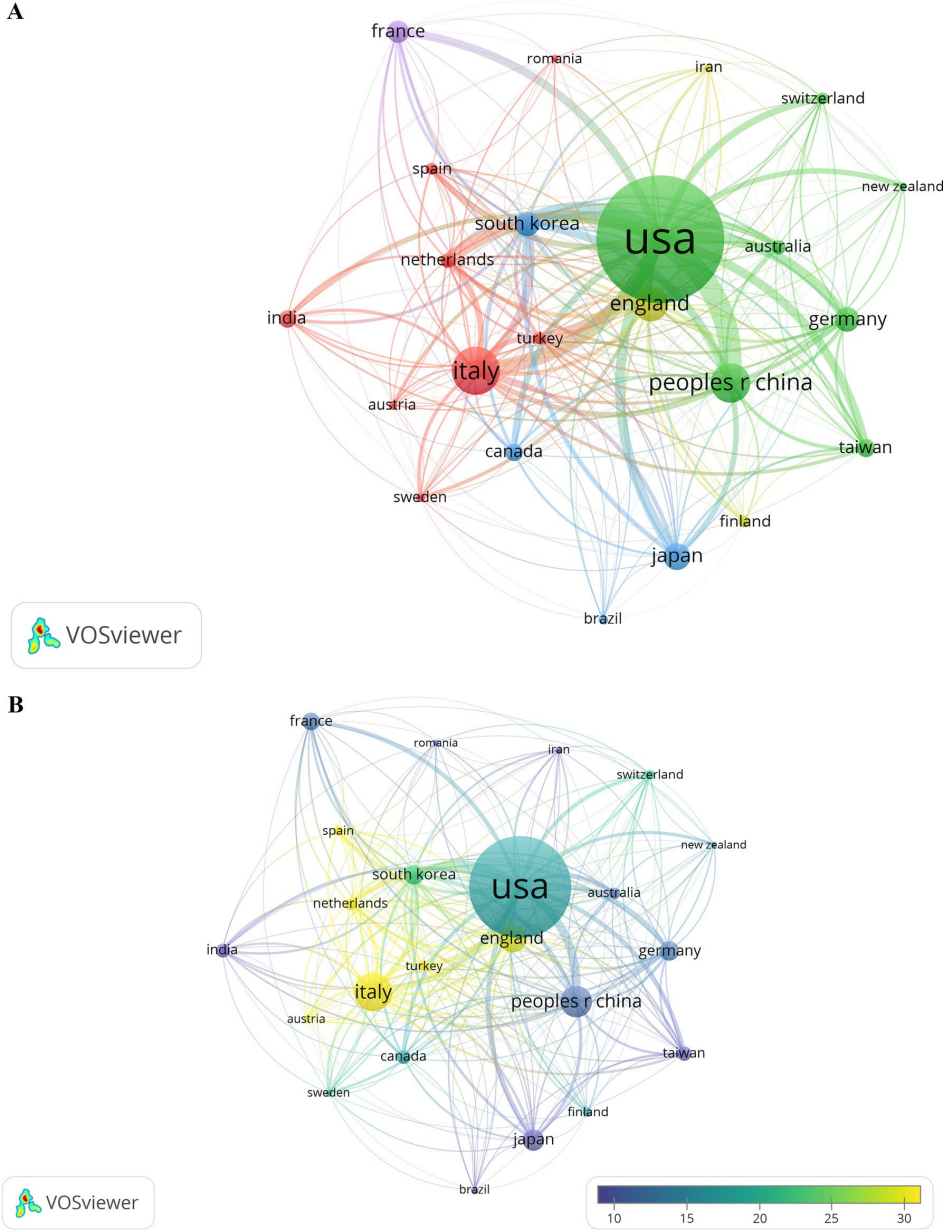

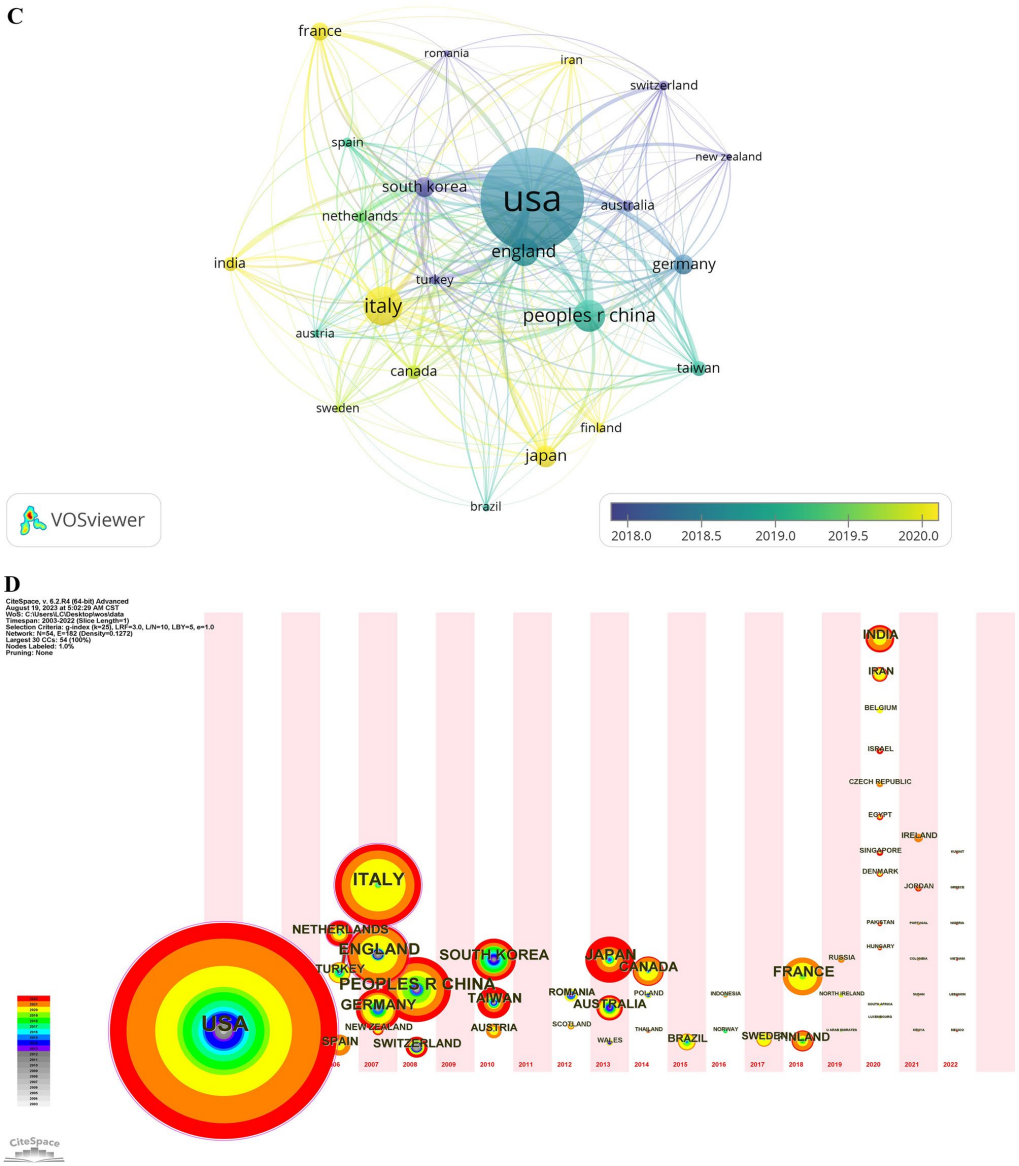

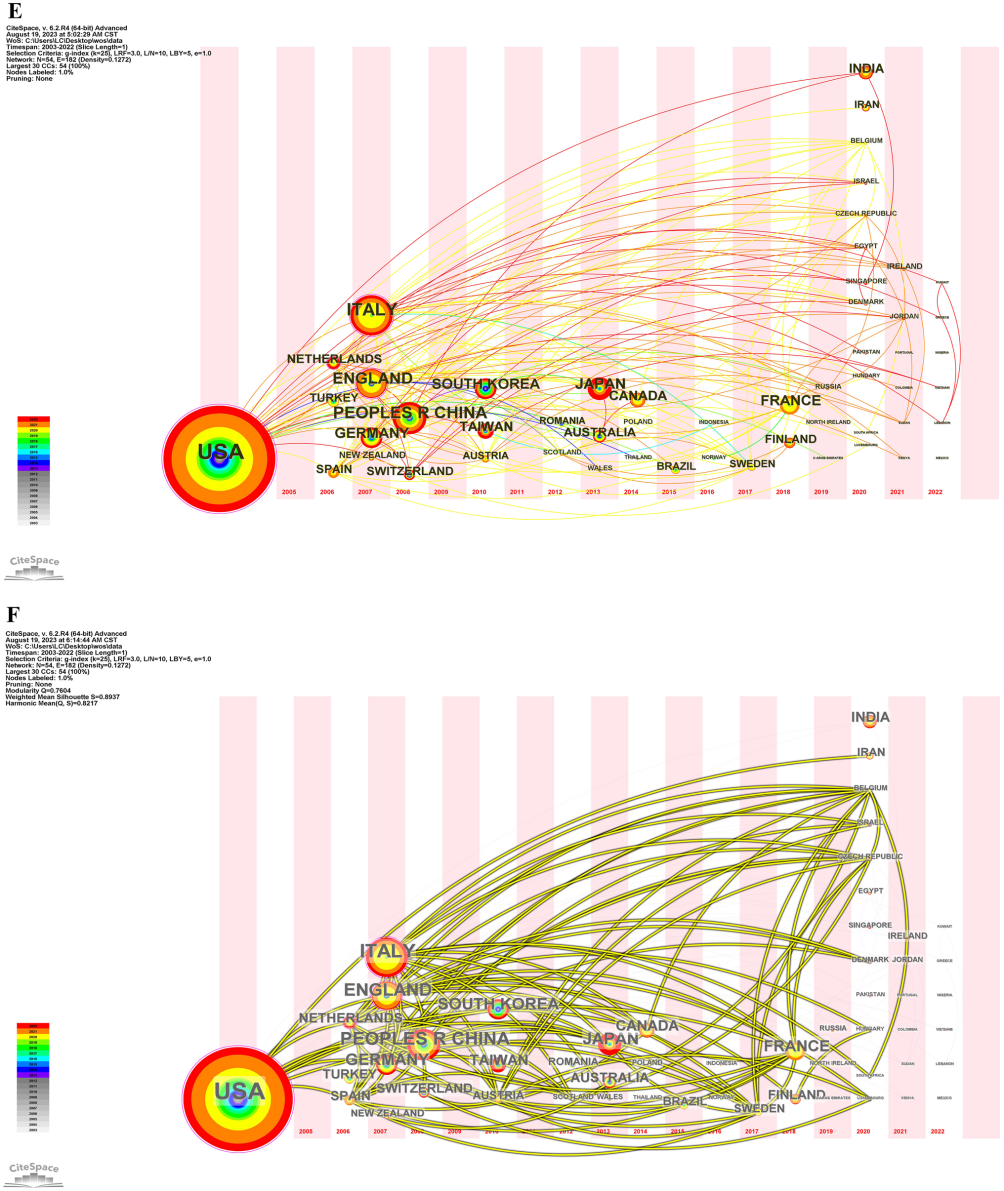
**A** Network visualization between countries/regions. The cluster of different color is determined by co-citing relationship. The closer two countries/regions are located to each other, the stronger their relatedness is. The strongest co-citation links between are represented by line. The higher the weight of an item, the larger the label and the circle of the item. **B** Average citation of countries/regions. The colors denote the average citation number in the right lower bar. **C** Average publish year of different countries/regions. Color of the circle denote the average publish year as in the right lower bar. **D** Timeline view of 54 countries’/regions’ research time and publication number. Circles appeared in each timeline means first research beginning time. The bigger the circle, the lager the citation number. Colors of rings means citations in different year from white to red as in the left bar. **E** Timeline view of countries’/regions’ co-citation. Colors of lines means co-citations of two countries/regions in different year from white to red as in the left bar. **F** Countries’/regions’ co-citation in 2020 (Color figure online)

A total of 363 papers are from 54 countries/regions. We select the top 11 published countries/regions as are shown in Table 2. USA published the most articles (*n* = 103), followed by Italy (*n* = 39) and China (*n* = 32). These three countries published nearly 50% of all the papers in the past 20 years. Meanwhile, the top 11 countries/regions covered more than 80% of all the 363 papers. 23 countries/regions are selected in the condition that publication is limited to “at least 5.” Figure 4A–C are created by VOSviewer. From Fig. 4A, we could easily find that USA played an important role among all the countries/regions appeared in this field. It actually ranked first in publication, followed by Italy and China. USA also had a highest co-citation linkage to China with link strength 128. Italy ranked second in publication but came first in average citation and followed by England shown in yellow from Fig. 4B. Average publish year is also counted out, with nearly half of the top 11 countries late than 2019 shown orange to red in Fig. 4C. Figure 4D–F are created by CiteSpace. They give another perspective about publication and citation. Figure 4D is a timeline about publication. In 2003, USA was the earliest country in studying surgical smoke, 3 years later in 2006, Spain, Netherlands, and Turkey consecutively published their study. Each circle means a country or region. The colorful annual rings denote publication number added in each year. USA published 26 papers, the most publication in 2020, defined the biggest orange ring in the chart. Figure 4E is a co-citation review. We could tell that most color of lines was close to red and connection became more intense. In fact, 2020 was a bursting year—the most articles (*n* = 92) were published, meanwhile, 12 countries published their first articles (*n* = 44), creating the biggest link net of countries’ co-citation as in Fig. 4F.

**Table 2:** Top 11 countries’/regions’ publication and citation of surgical smoke

Rank	Country/region	Number of publication	%Of (363)	Citations	Average citation	Average publish year
1	USA	103	28.37%	1764	17.13	2018.53
2	Italy	39	10.74%	1359	34.85	2020.08
3	Peoples R China	32	8.82%	388	12.13	2018.94
4	England	28	7.71%	806	28.79	2018.68
5	Japan	22	6.06%	209	9.50	2020.32
6	Germany	20	5.51%	260	13.00	2018.4
7	South Korea	20	5.51%	448	22.40	2017.6
8	France	18	4.96%	229	12.72	2020.06
9	Taiwan	15	4.13%	155	10.33	2018.93
10	Canada	14	3.86%	240	17.14	2019.79
11	India	14	3.86%	49	3.50	2020.86

### Funding agencies analysis

A total of 217 funding agencies published articles of surgical smoke. Table 3 showcases the top 16 most distributed funding agencies of surgical smoke. National Natural Science Foundation of China ranked first with 8 articles, followed by Tampere Tuberculosis Foundation (*n* = 7) and National Institute for Occupational Safety and Health (*n* = 6). Finnish funding agencies played a highlighted part in this field. There are 7 funding agencies of Finland appeared in this table, followed by USA (4 funding agencies included). China and Switzerland both have 2 agencies on the list while Korea has 1.

**Table 3:** Top 16 funding agencies of surgical smoke

Funding agency	Number
National Natural Science Foundation of China	8
Tampereen Tuberkuloosisaatio (Tampere Tuberculosis Foundation)	7
National Institute for Occupational Safety and Health	6
Finnish Foundation for Technology Promotion (TES)	5
National Research Foundation of Korea	5
ETH Zurich	4
Emil Aaltonen foundation	4
Academy of Finland	3
Doctoral School of Tampere University	3
Finnish Cultural Foundation	3
Finnish Medical Foundation	3
Ministry of Science and Technology	3
NIOSH	3
Science and Technology Project of Zhejiang Province	3
Swiss National Science Foundation	3

### Analysis of authors

**Fig. 5:**
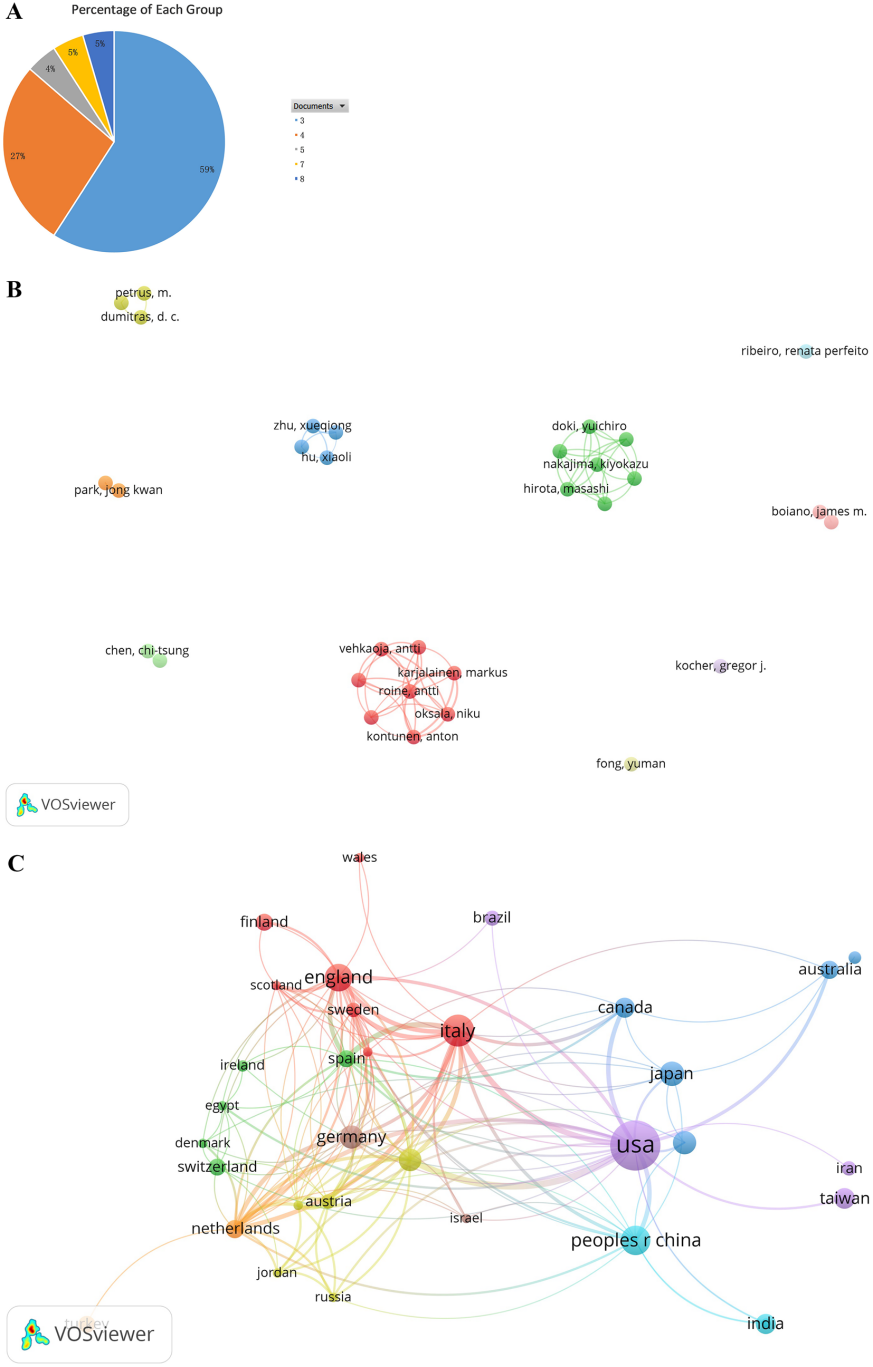
**A** Percentage of documents from five publishing groups. **B** Authors’ network visualization of co-authors. **C** Countries’/regions’ network visualization of co-authors

There are 1847 authors for these 363 articles between 2003 and 2022. We get 44 authors (Table 4) and their 166 articles on surgical smoke after changing “the minimum number of documents of an author” to 3. Among them, there are 2 authors publishing the most papers (*n* = 8), 2 authors of 7 papers, 2 authors of 5 papers, 12 authors of 4 papers, and 26 authors of 3 papers. All the articles are divided into five groups based on the author’s publication. The articles in 3-paper-group occupies more than half in these selected 166 articles, shown in Fig. 5A. Among the top 6 most published authors, 5 authors come from Finland, 1 from China. Citations are extracted as well, Zhu, Xueqiong has the most citations (*n* = 143), followed by Hu, Xiaoli (*n* = 138) and Choi, Seock Hwan (*n* = 121), Zhu and Hu are Chinese while Choi are Korean. Co-authorship is analyzed in two different ways by authors and countries. Figure 5B shows authors-based co-authorship, in which there are a few scattered collaborations, and authors are fixed to certain group, no group–group linkages seen. Likewise, countries-based co-authorship is presented in Fig. 5C. The picture tells that good international cooperation has been keeping between authors. Inspiringly, USA, Italy, and Netherlands have maintained collaboration relationship with more than 20 countries/regions. It is still expecting to see teamwork on surgical smoke between more authors, deeply and massively.

**Table 4:** Authors of no less than three papers and document count and citations

Author	Document count	Citations
Oksala, Niku	8	91
Roine, Antti	8	91
Karjalainen, Markus	7	86
Kontunen, Anton	7	86
Vehkaoja, Antti	5	17
Zhu, Xueqiong	5	143
Anttalainen, Anna	4	10
Anttalainen, Osmo	4	10
Doki, Yuichiro	4	29
Gianella, Michele	4	60
Hirota, Masashi	4	29
Mauro, Alessandro	4	34
Nakajima, Kiyokazu	4	29
Park, Jong Kwan	4	42
Sigrist, Markus W.	4	60
Soo, Jhy-Charm	4	86
Takahashi, Hidekazu	4	29
Yamasaki, Makoto	4	29
Boiano, James M.	3	86
Chen, Chi-Tsung	3	13
Choi, Seock Hwan	3	121
Dumitras, D. C.	3	15
Fong, Yuman	3	94
Hu, Xiaoli	3	138
Kim, Fernando J.	3	30
Kocher, Gregor J.	3	27
Kumpulainen, Pekka	3	7
Kurokawa, Yukinori	3	10
Lacey, Steven E.	3	59
Lee, Sang Kyi	3	39
Lippert, Julia F.	3	59
Liu, Yi	3	91
Lopez, Ramon	3	59
Massarotti, Nicola	3	27
Molina, Wilson R.	3	30
Patachia, M.	3	15
Petrus, M.	3	15
Ribeiro, Renata Perfeito	3	18
Sehrt, David	3	30
Steege, Andrea L.	3	86
Takahashi, Tsuyoshi	3	10
Vortman, Rebecca	3	4
Wan, Gwo-Hwa	3	13
Yan, Linzhi	3	91

### Analysis of keywords

**Fig. 6:**
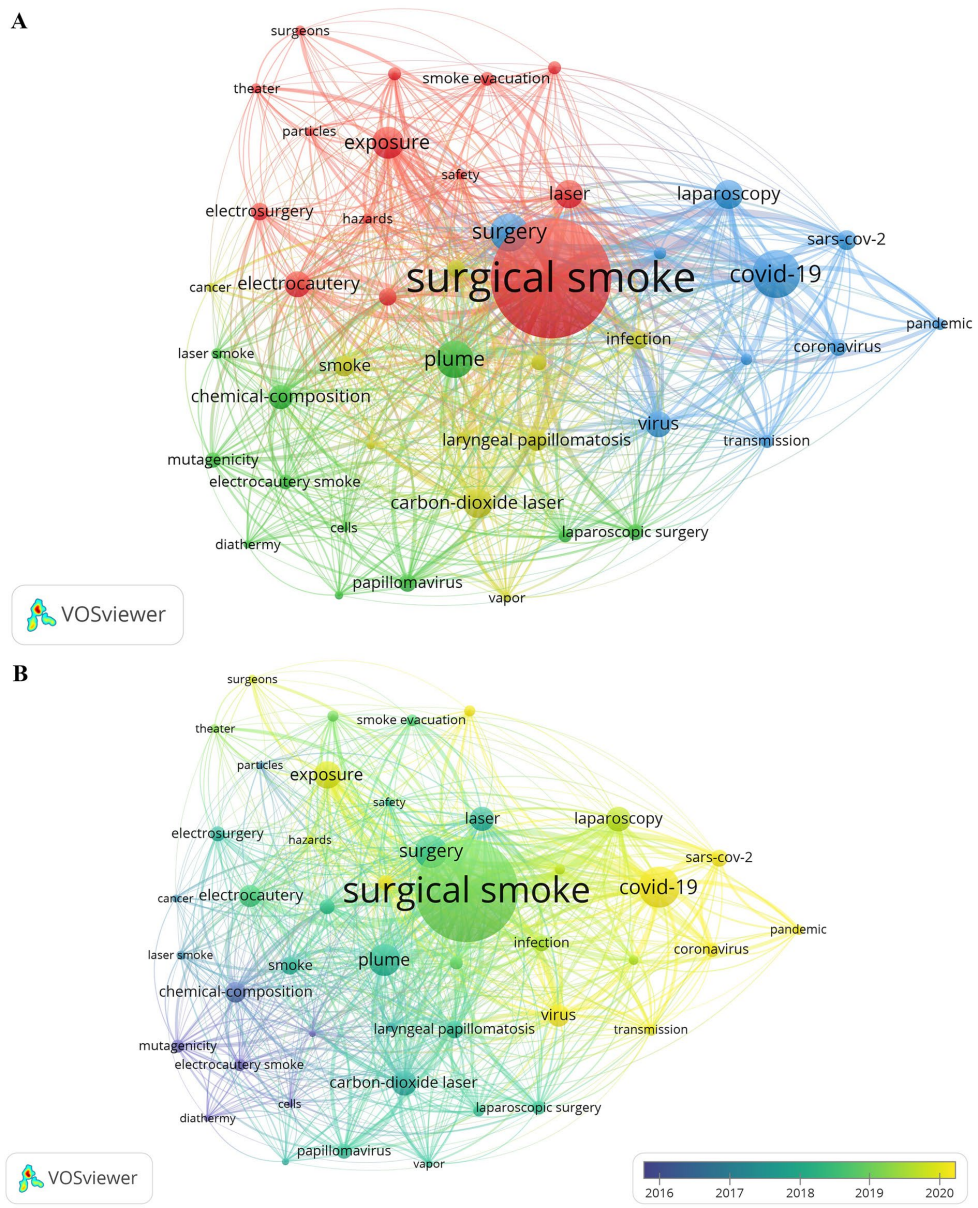

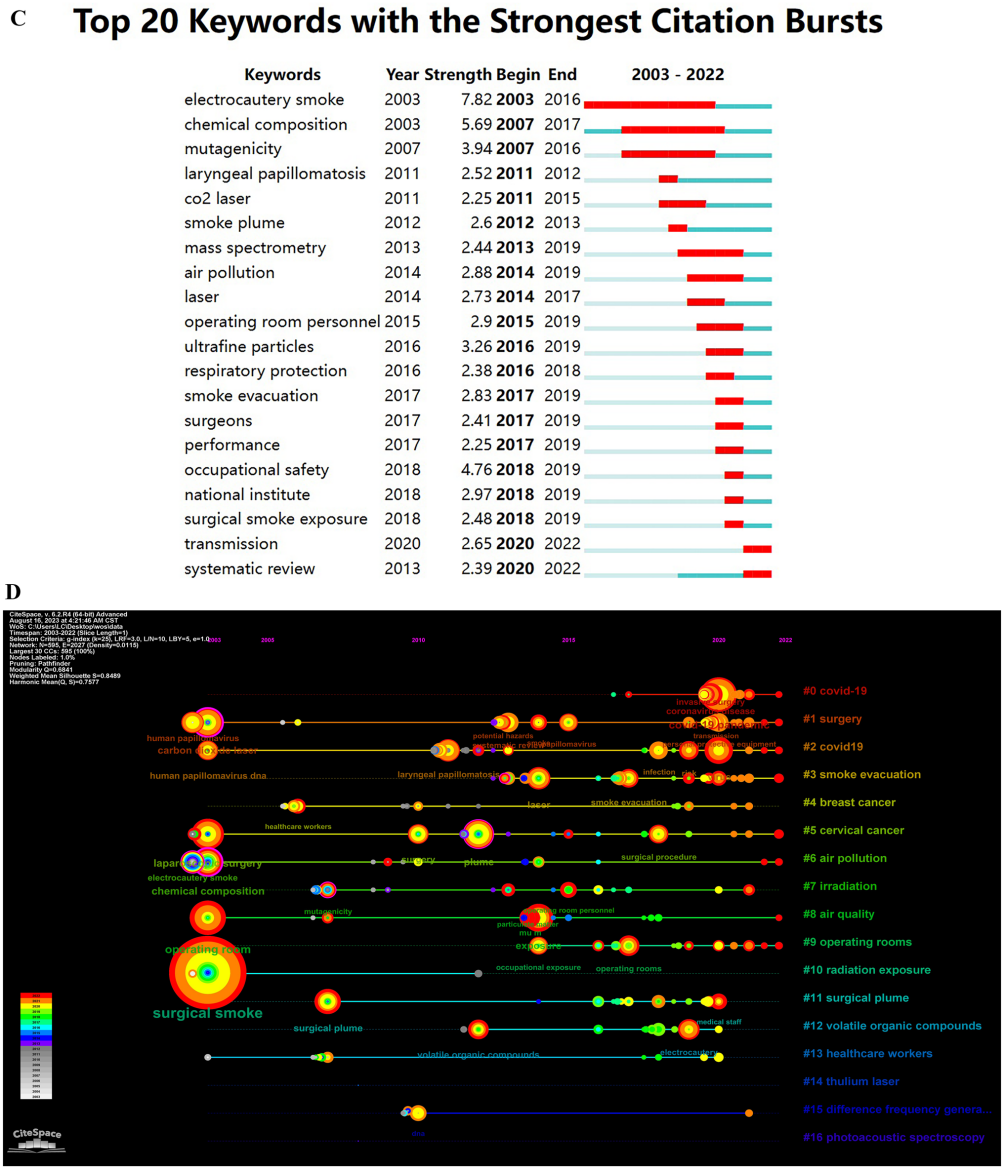
**A** Network analysis of keywords co-occurrence. **B** Co-occurrence analysis of key words and time superposition. **C** Top 20 keywords of strong citation burst. **D** Terms from keywords cited more than 20 times. One circle means a keyword, inside which the color and size of the ring denote the citation in a year shown in the left bar. The colored noun phrases in right side denotes the theme in the year shown in the left bar (Color figure online)

A total of 1321 keywords were extracted, among which 96 keywords appeared at least 5 times, 45 keywords appeared at least 10 times, and 24 appeared at least 20 times. We extracted the top 20 appeared keywords in Table 5. “Surgical smoke” (*n* = 230) ranked first, followed by “covid-19” (*n* = 79) and “surgery” (*n* = 61). Figure 6A and B are based on the keywords appeared more than 10 times. The line between two circles means co-occurrence in papers, and thickness of the line is positively relative to the link strength. In Fig. 6A, red cluster denotes the management about surgical smoke; green cluster is the physico-chemical influence, while yellow and blue clusters are about the biological hazard. Figure 6B shows the gradually change of hot-spot keywords, and the latest hot-spot keywords is “COVID-19,” “virus,” “transmission,” “exposure,” and “risk,” taking the place of “laparoscopy,” “infection,” and “smoke evacuation.” Figure 6C is a top 20 keywords of strong citation burst. “Electrocautery smoke” has a longest citation burst (*n* = 13), followed by “chemical composition” (*n* = 10) and “mutagenicity” (*n* = 9). Nevertheless, “transmission” and “systematic review” are latest appeared and still lasting in citation burst. Figure 6D, created and counted by CiteSpace, gives us an annual vision on keywords. All the 17 terms are deduced by the software indicate different themes in the last 20 years. “Covid-19,” “cancer,” “surgery,” and “smoke evacuation” stand for the hot spots in the past 5 years.

**Table 5:** Co-occurrence and link strength of the top 20 keywords

Rank	Keyword	Occurrences	Total link strength
1	Surgical smoke	230	781
2	COVID-19	79	362
3	Surgery	61	257
4	Plume	60	345
5	Exposure	50	218
6	Carbon-dioxide laser	44	300
7	Laparoscopy	44	212
8	Laser	43	199
9	Electrocautery	39	203
10	Virus	39	252
11	Chemical-composition	37	187
12	Laryngeal papillomatosis	30	179
13	Smoke	30	144
14	SARS-CoV-2	29	144
15	Infection	26	164
16	Electrosurgery	25	87
17	Papillomavirus	25	142
18	Risk	25	143
19	Surgical plume	25	98
20	Coronavirus	24	114

### Co-cited references and reference burst

**Fig. 7:**
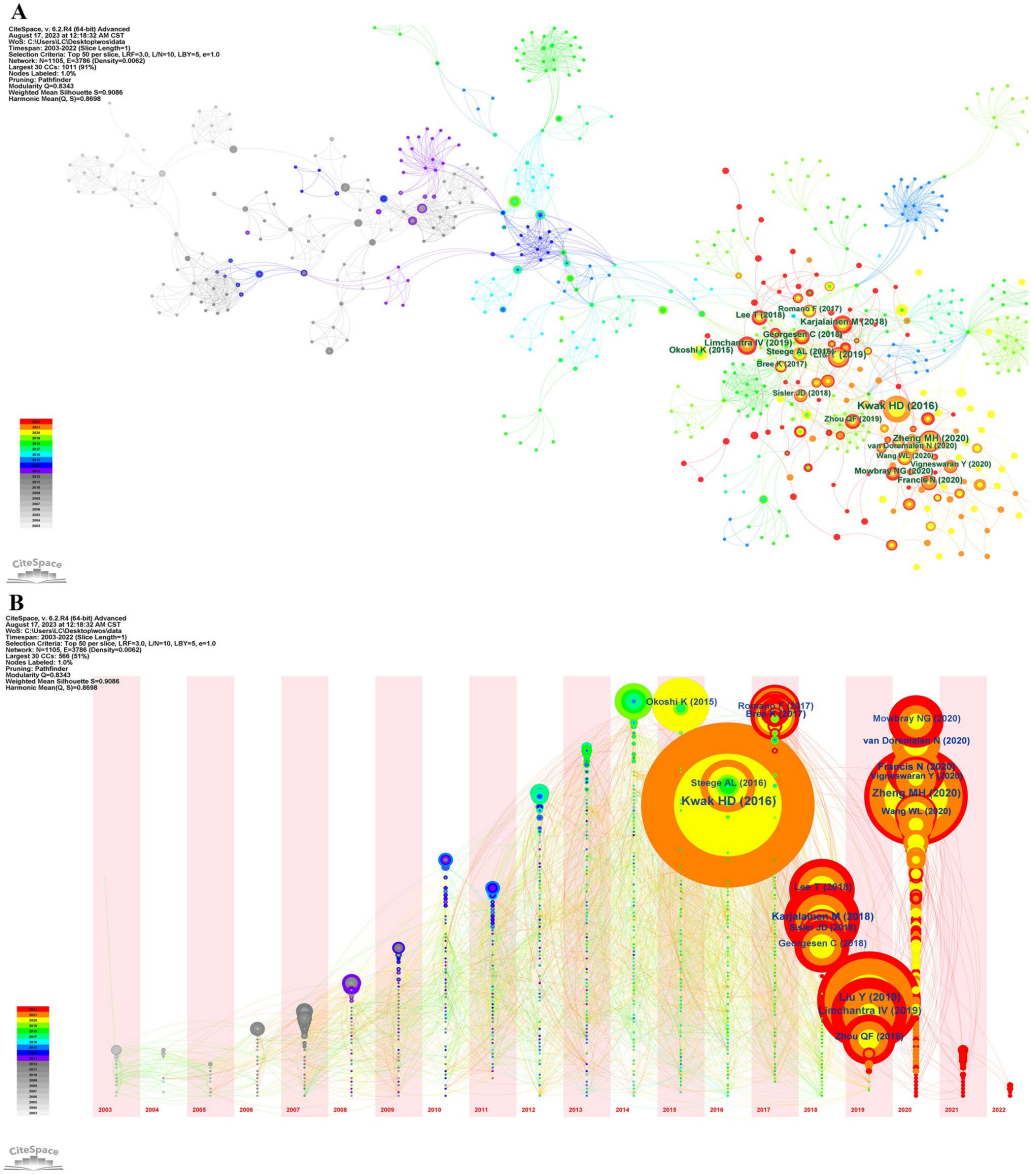

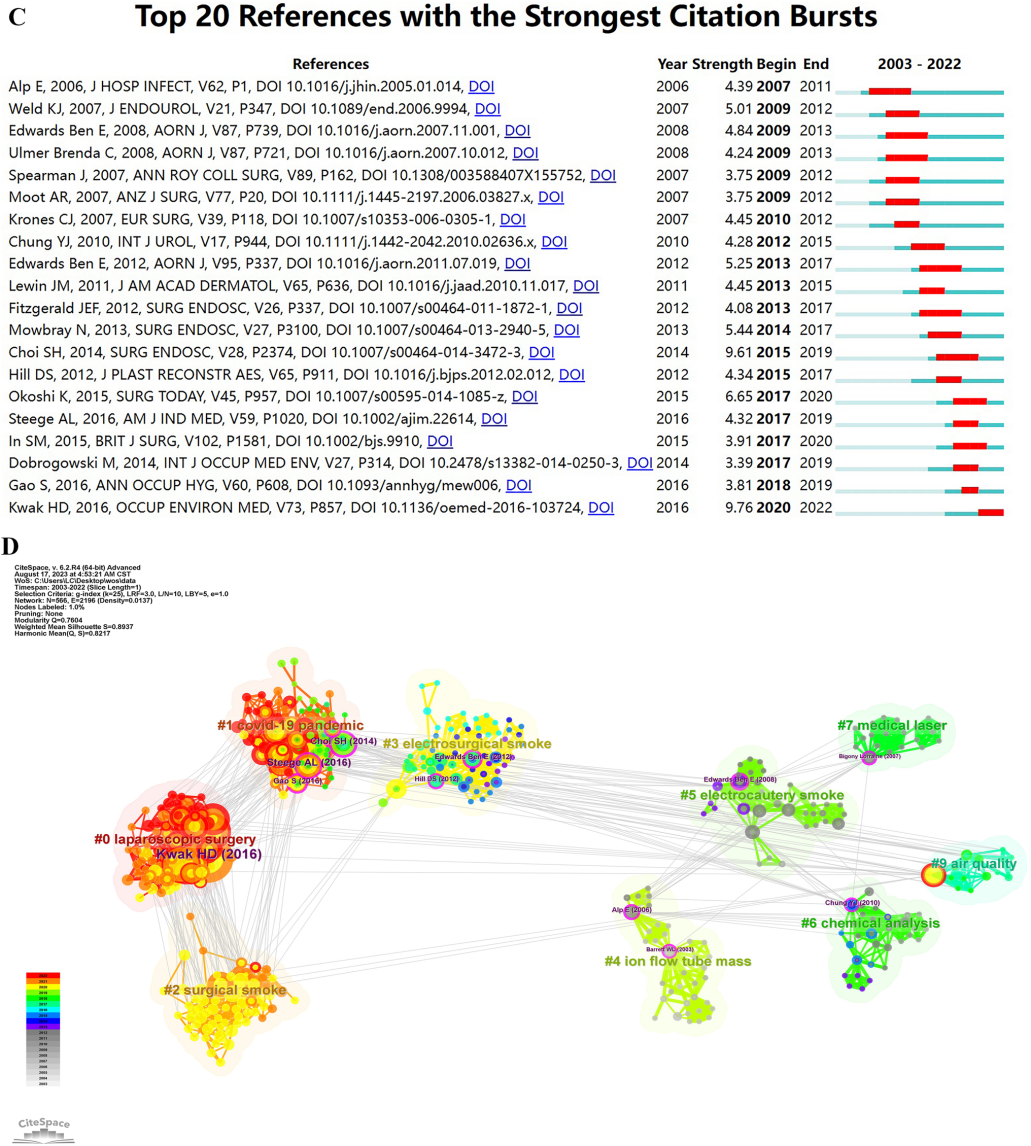
**A** The references network visualization. **B** The timezone view of references network. Circles with blue label are references co-cited more than 20 times. Colors of rings means citation year from white to red as in the left bar, and the size of the ring means citation number. **C** The references with strong citation bursts. **D** Clusters generated by the titles of references. Color of the clusters denote the trend on surgical smoke from 2003 to 2022. Purple ring in the outer layer denote the bridge references between clusters (Color figure online)

Based on the data from CiteSpace, there are 1105 co-cited references and 3786 links we obtained in all 363 articles. Among them, 38 references are cited more than 10 times, while 18 references are cited more than 20 times shown in Fig. 7A and B. All these 38 references are published later than 2013 and more specifically, 20 of them are published later than 2019. The most co-cited article is Han Deok Kwak authored “Detecting hepatitis B virus in surgical smoke emitted during laparoscopic surgery” published on Occupational and environmental medicine. It even has a frequency of up to 47 co-citations in 2020 due to the affecting characteristics of COVID-19 virus. We listed the top 10 co-cited reference in Table 6 on surgical smoke. Topics like “COVID-19,” “surgical smoke hazard,” and “surgical smoke evacuation or prevention” are included in these references. From Fig. 7C, the strongest citation bursts are shown red in the right blue bar. As time went by, the citations of older references became fewer and fewer, which could be explained by knowledge updating. The difference is that most of the references have had its citation burst in 2 years and ended after 5-year publication, but the reference from Kwak HD came to its burst citations after 3 years and didn’t meet its end after 6-year publication. This result matches to and complementarily explains what has showed in Fig. 7A and B. All data are analyzed to created a cluster trend network and selected the top 10 clusters as in Fig. 7D, based on the titles of references by CiteSpace. This picture tells us the hot-spot trend in the past 20 years on surgical smoke. Of all the clusters, “laparoscopic surgery” ranked first, with “COVID-19 pandemic” and “surgical smoke” as second and third. Circle with purple rings in the outer layer denote crucial references between clusters. Reading these articles might be helpful to know the macroscopic trend view on surgical smoke in the latest 20 years. All the relative references have been listed in Table 7.

**Table 6:** Top 10 co-cited references on surgical smoke

Author	Title	Year	Journal
Kwak HD	Detecting hepatitis B virus in surgical smoke emitted during laparoscopic surgery	2016	Occup Environ Med
Zheng MH	Minimally invasive surgery and the novel coronavirus outbreak: lessons learned in China and Italy	2020	Ann Surg
Liu Y	Awareness of surgical smoke hazards and enhancement of surgical smoke prevention among the gynecologists	2019	J Cancer
Limchantra IV	Surgical smoke exposure in operating room personnel: a review	2019	JAMA Surg
Karjalainen M	The characterization of surgical smoke from various tissues and its implications for occupational safety	2018	PLoS ONE
Lee T	Surgical smoke control with local exhaust ventilation: experimental study	2018	J Occup Environ Hyg
Francis N	SAGES and EAES recommendations for minimally invasive surgery during COVID-19 pandemic	2020	Surg Endosc
Okoshi K	Health risks associated with exposure to surgical smoke for surgeons and operation room personnel	2015	Surg Today
Georgesen C	Surgical smoke: risk assessment and mitigation strategies	2018	J Am Acad Dermatol
Mowbray NG	Safe management of surgical smoke in the age of COVID-19	2020	Br J Surg

**Table 7:** Crucial references between clusters

Number	References
1	Barrett WL, Garber SM. Surgical smoke: a review of the literature. Is this just a lot of hot air? Surg Endosc. 2003 Jun;17(6):979–987. https://doi.org/10.1007/s00464-002-8584-5
2	Alp E, Bijl D, Bleichrodt RP, Hansson B, Voss A. Surgical smoke and infection control. J Hosp Infect. 2006 Jan;62(1):1–5. https://doi.org/10.1016/j.jhin.2005.01.014
3	Chung YJ, Lee SK, Han SH, Zhao C, Kim MK, Park SC, Park JK. Harmful gases including carcinogens produced during transurethral resection of the prostate and vaporization. Int J Urol. 2010 Nov;17(11):944–949. https://doi.org/10.1111/j.1442-2042.2010.02636.x. Epub 2010 Sep 29
4	Bigony L. Risks associated with exposure to surgical smoke plume: a review of the literature. AORN J. 2007 Dec;86(6):1013–1020; quiz 1021–1024. https://doi.org/10.1016/j.aorn.2007.07.005. PMID: 18068405
5	Edwards BE, Reiman RE. Results of a survey on current surgical smoke control practices. AORN J. 2008 Apr;87(4):739–49. https://doi.org/10.1016/j.aorn.2007.11.001
6	Edwards BE, Reiman RE. Comparison of current and past surgical smoke control practices. AORN J. 2012 Mar;95(3):337–350. https://doi.org/10.1016/j.aorn.2011.07.019
7	Hill DS, O’Neill JK, Powell RJ, Oliver DW. Surgical smoke—a health hazard in the operating theatre: a study to quantify exposure and a survey of the use of smoke extractor systems in UK plastic surgery units. J Plast Reconstr Aesthet Surg. 2012 Jul;65(7):911–916. https://doi.org/10.1016/j.bjps.2012.02.012
8	Choi SH, Kwon TG, Chung SK, Kim TH. Surgical smoke may be a biohazard to surgeons performing laparoscopic surgery. Surg Endosc. 2014 Aug;28(8):2374–2380. https://doi.org/10.1007/s00464-014-3472-3. Epub 2014 Feb 26
9	Steege AL, Boiano JM, Sweeney MH. Secondhand smoke in the operating room? Precautionary practices lacking for surgical smoke. Am J Ind Med. 2016 Nov;59(11):1020–1031. https://doi.org/10.1002/ajim.22614
10	Shuang Gao et al., Performance of facepiece respirators and surgical masks against surgical smoke: simulated workplace protection factor study, The Annals of Occupational Hygiene, Volume 60, Issue 5, June 2016, Pages 608–618, https://doi.org/10.1093/annhyg/mew006

## Discussion

In this study, we retrieved 363 eligible publications from 54 countries or regions in WoSCC on surgical smoke with time limited in 20 years (from January 1st 2003 to December 31st 2022). VOSviewer, CiteSpace, and Excel 2019 were used to collect and visualize the data to present the dynamic change and research trend in surgical smoke. Bibliometric analysis was performed in journals, countries/regions, funding agencies, authors, key words, and references.

It is found that the yearly publication number and citation has increased in a low speed until a big surge occurred in 2020, a bursting year proved to reach the peak both in the publication and citation during the past 20 years. No doubt this phenomenon has something to do with the COVID-19 Pandemic. COVID-19 broke out at the year end of 2019, then instantly but extensively transmitted worldwide in 2020, and caught incomparably supreme attention of medical staff, epidemiological specialist, and even human beings on the earth. Many scholars from different regions turned to the studies about prevention and protection against COVID-19. In the field of surgical smoke, 230 papers have been published in the latest 3 years (between 2020 and 2022), compared with 133 papers published in previous 17 years (from 2003 to 2019). Still, the former is nearly twice more than the latter.

In all the countries or regions, USA has made the greatest contribution to surgical smoke, with the biggest number both in publication and citation. It is also the earliest and continuously researching country in this field. As the second most published and cited country, Italy owns the highest scores in average citation by its outstanding performance in publication after 2020. China, ranking the third in publication, has the most published funding agency: National Natural Science Foundation of China. Meanwhile, Finland, began its research at 2018, though, had 7 funding agencies with over three papers, and consequently ranked first in terms of funding agency number. As more countries joined in this field since 2020, more studies and co-operations will be performed to acquire new material or better equipment, and eventually eliminate hazard from surgical smoke in the future.

In the aspect of authors, Roine, A and Oksala, N, who worked together in the same Finnish group with Karjalainen, M; Kontunen, A and Vehkaoja, A, are both the most published authors. In their study, they verified there were significant differences in particles of surgical smoke when electrosurgical devices worked on different tissues. Liver produced the most particles, followed by renal tissues and skeletal muscle, while fat, lung tissue, cerebral gray and white matter, and skin produced considerably less particulate mass [30]. What’s more, they introduced Differential Ion Mobility Spectrometry (DMS) and invented Automatic Tissue Analysis System (ATAS) to differentiate tissues originated from ten different organs and discriminate benign and malignant tissues based on surgical smoke, which might help clinical surgeons with margin assessment in the future [31–33]. When it comes to citation, Zhu, Xueqiong, who worked together with Hu, Xiaoli in China, ranks first by surgical smoke among gynecologists. They proved the prevalent presence of HPV DNA in surgical smoke during cervical operation, moreover, HPV DNA sampled from surgeon’s nasal epithelial was tested positive, though not infected [6, 34]. This study echoed and explained the suspicious HPV-infectious two carcinoma cases happened to male gynecologists [35].

The latest hot spot key words is about “COVID-19” and “virus,” while “surgical smoke evacuation” is of great importance to prevent occupational exposure in surgery. The study trend might be about “surgical smoke” during “laparoscopic surgery” in the background of COVID-19. The most co-cited reference is “Detecting hepatitis B virus in surgical smoke emitted during laparoscopic surgery” [36], written by Kwak HD and published on OCCUPATIONAL AND ENVIRONMENTAL MEDICINE in 2016. The team collected a median volume gas of 375 L, with a high-efficient collector named Biosampler attached to the 5 mm trocar outlet, then the samples were transferred to laboratory in less than 1 h to get analyzed by using nested PCR. 10 of the 11 cases discovered HBV in surgical smoke. This is the first article about HBV isolation from surgical smoke of laparoscopy, which provide a feasible method to detect virus in a virus-infection surgery. Based on the potentially risky characteristics that virus could be transmitted through mucosal membrane into circulatory system, scholars adopted comprehensive measures to prevent the novel coronavirus from contaminating operating room, as in “Minimally Invasive Surgery and the Novel Coronavirus Outbreak: Lessons Learned in China and Italy” [37], the second most co-cited article, which told us to pay attention to the professionals’ health and occupational safety besides patients’. While the third most co-cited article, “Awareness of surgical smoke hazards and enhancement of surgical smoke prevention among the gynecologists” gives us a relatively overall description about surgical smoke with protective measures included.

From the most co-cited and crucial references, we conclude four ways to minimize the risk of surgical smoke: Firstly, use wall suction with an in-line filter. Evacuating surgical smoke with wall suction is common practice, but the shortcoming is that the smoke might cycle into environment anywhere. So, wall suction with an in-line filter is recommended, which is convenient and adequate for many surgeries [38]. The distance should be within 2–3 cm from smoke source for effectiveness [39]. It can also be used in laparoscopic surgery to preventing smoke built up by attaching to an partially opened trocar [40]. Secondly, apply smoke evacuator with filter. This method can be used in procedure generating larger volume of smoke [41]. Additionally, there has been laparoscopy-used device with filter inside to evacuate automatically when smoke is detected, which proved effective for surgical smoke [13]. Thirdly, personnel protection equipment should be required. The high efficiency particulate air (HEPA) filter, like N95 respirator, ought to be used for respiratory protection when necessary. Surgical masks can filter the vast majority of noxious chemicals in surgical smoke [22] but can not block particles less than 5 μm. In another word, mutagenic, carcinogenic gases, or viable biologic particles, if less than 5 μm, could be breathed in [40]. While N95 respirator could filter over 95% 0.3-μm-sized particles, and reduce the inhalation exposure to surgical smoke by over two orders of magnitude [42]. Disadvantage is that wearers may suffer from respiratory discomfort due to the tight closure and high-efficient filtration. At last, more efforts should be made in further training for surgical teams about surgical smoke, in approach of curriculum, examination or short video, in order to raise awareness of collective occupational security, work together and reduce occupational exposures [22, 40, 41].

### Strengths and limitations

This study is the first one, to our knowledge, that comprehensively describe the document characteristics and study trends about “surgical smoke” by bibliometrics. Additionally, two kinds of widely used bibliometric software were employed to create document maps and visualize research data in each way, objectively showing the trends and hot spots and giving readers a general idea about surgical smoke. Nevertheless, this study have limitations. First, for the analysis of the bibliometric software, all articles were retrieved merely from WoSCC and the language was restricted to only English, therefore, certain important studies collected in other databases or in other languages might be omitted; secondly, we only selected data published in the last 20 years, some classic literature could not be covered, leading to certain biased results.

## Conclusion

According to bibliometric analysis, the research on surgical smoke is drawing attention of more scholars in the world. Increasing number of countries or regions added in this field, and among them, USA, Italy, and China are playing important roles, however, more wide and intense cooperation is still in expectation. Particularly, under the influence of COVID-19, biological adverse of surgical smoke has been up to the premiere hotspot. With more studies that prove virus exists in surgical smoke, plus previous studies that chemical matters in surgical smoke are hazardous to human, there are more studies on protection or prevention from surgical smoke, but effective and simple measures or devices might still need further developing and examining.

### Supplementary Information

Below is the link to the electronic supplementary material.

Supplementary file1 (TXT 2087 kb)

Supplementary file2 (XLSX 631 kb)
