# Correction: Knowledge mapping of surgical smoke from 2003 to 2022: a bibliometric analysis

**DOI:** 10.1007/s00464-024-10707-z

**Published:** 2024-02-01

**Authors:** Chuang Li, Meng Geng, Shujun Li, Xianglan Li, Huiqin Li, Hufang Yuan, Fengxia Liu

**Affiliations:** 1 The Fourth Hospital of Hebei Medical University, Shijiazhuang, China; 2 Hebei Medical University Third Hospital, Shijiazhuang, China

**Correction to: Surgical Endoscopy** 10.1007/s00464-023-10641-6

The original online version of this article was revised to correct the presentation of the authors' affiliations. The affiliations are as follows:

Affiliation 1: The Fourth Hospital of Hebei Medical University, Shijiazhuang, China

Affiliation 2: Hebei Medical University Third Hospital, Shijiazhuang, China

The original article has been corrected.

